# COMPARATIVE EVALUATION OF SKIN SUTURE IN RATS WITH POLYGLYCAPRONE 25 AND NYLON

**DOI:** 10.1590/1413-785220233104e266635

**Published:** 2023-07-31

**Authors:** RAFAEL SALEME ALVES, LEONARDO YABU TANAKA, VICTOR BIGNATTO CARVALHO, LETICIA CANDIDO LOPES, SOFIA BRANDÃO DOS SANTOS, NUHA AHMAD DSOUKI, BRUNO FIORELINI PEREIRA, MONICA AKEMI SATO

**Affiliations:** 1 Centro Universitario Faculdade de Medicina do ABC, Departamento de Ortopedia e Traumatologia, Santo Andre, SP, Brazil.; 2 Centro Universitario Lusiada, Faculdade de Ciencias Medicas de Santos, Santos, SP, Brazil.; 3 Centro Universitario Faculdade de Medicina do ABC, Departamento de Morfologia e Fisiologia, Santo Andre, SP, Brazil.; 4 Universidade Federal de Sao Paulo, Departamento de Ciencias Biologicas, Diadema, SP, Brazil.

**Keywords:** Skin, Wound Healing, Sutures, Inflammation, Metalloproteinases, Tissue Inhibitor of Metalloproteinases, Pele, Cicatrização, Suturas, Inflamação, Metaloproteinases, Inibidores Teciduais de Metaloproteinases

## Abstract

Currently, the market offers a wide variety of suture threads, made of materials with different structural and chemical properties. Among many other characteristics, they vary in origin, absorption or degradation, and structure. From this variety, the clinical doubt arises as to which material provides the patient with the best healing quality.

**Objective:**

This study aims to comparatively evaluate two different types of suture threads-Monocryl^®^ (polyglycaprone 25) and Ethilon^®^ (nylon)-regarding their ability to aid in tissue regeneration by a histological and immunohistochemical analysis of the skin of rats sutured with the aforementioned materials.

**Methods:**

This basic experimental study used 12 adult Wistar rats, randomly divided into three groups with four animals each and subjected to four longitudinal incisions under anesthesia. Each group corresponded to a postsurgical evaluation date (one, seven, and 14 days).

**Results:**

At 14 postoperative days, the studied groups had no histological difference. However, the use of nylon thread showed greater evidence of earlier fibrotic union.

**Conclusion:**

This study found no histological difference in healing 14 days after surgery among the techniques and the types of suture threads. ***Level of Evidence II, Therapeutic Studies.***

## RESUMO

Atualmente, encontra-se disponível no mercado uma grande variedade de fios de sutura, compostos de materiais com diferentes propriedades estruturais e químicas, que variam quanto à origem, absorção ou degradação e estrutura, entre outras características. A partir dessa disponibilidade, emerge a dúvida clínica quanto ao material que propicia a melhor qualidade de cicatrização ao paciente.

**Objetivo:**

Avaliar comparativamente dois tipos de fios - Monocryl^*®*^ (poliglicaprone 25) e Ethilon^*®*^ (nylon) - quanto à sua capacidade de auxílio na regeneração tecidual, por meio da análise histológica e imuno-histoquímica da pele de ratos submetidos a suturas com esses materiais.

**Métodos:**

Neste estudo básico experimental, foram utilizados 12 ratos adultos da linhagem Wistar, randomicamente divididos em três grupos com quatros animais cada, que foram submetidos a quatro incisões longitudinais sob anestesia. Cada grupo correspondeu a uma data de avaliação pós-cirúrgica (1, 7 e 14 dias).

**Resultados:**

Passados 14 dias após a operação, não houve diferença histológica em relação aos grupos estudados. No entanto, o uso de fio de nylon apresentou evidência de união fibrótica mais precoce.

**Conclusão:**

Não há diferença histológica de cicatrização após 14 dias pós-operatórios entre as técnicas e os tipos de fio de sutura. **Nível de Evidência II, Estudos Terapêuticos.**

## INTRODUCTION

Synthesis is the last step of a surgical procedure and, compared with other methods used at this stage, such as adhesives, suturing is the most frequent, which makes suture materials the most common exogenous implants found in human organisms.^1^ The main functions of the suture include promoting closure and healing of the wound or surgical incision, and helping reduce possible infections by restoring continuity between the edges and layers separated in the dieresis.^2^

Each material has a distinct set of structural and chemical properties, which interferes with its ability to prevent infection, minimize inflammation, and aid in the healing process. Among the aforementioned factors, inflammation is an inherent response of the body to the implantation of threads, which are interpreted as foreign bodies. Normally, sutures of animal origin have greater inflammatory tissue reactions.

The use of materials with greater tensile strength and suture firmness also implies the search for materials with less inflammatory response, such as monofilaments.^3^ ^)-(^ ^5^ Nylon, in its monofilament form, causes little tissue reaction and can be used and well tolerated in infected tissues. Similarly, another monofilament suture, Monocryl (absorbable, epsilon-caprolactone, and glycolide copolymer), is easy to handle and has minimal resistance during tissue passage and adequate tension. The absorption time is completed about 120 days after implantation in the tissue, with minimal tissue reaction.^6^

The suture technique applied is also important in the progress of wound closure and healing. In 2014, Gurusamy et al.,^7^ in a review comparing five studies on continuous sutures with interrupted skin sutures for 730 participants undergoing nonobstetric operations, found no significant difference in the proportion of participants who developed superficial site infection between the continuous suture and interrupted suture groups. A total of 23 participants (23/625; 3.7%) developed superficial wound dehiscence. Among the 23, 22 participants were part of the interrupted suture groups. The proportion of participants who developed superficial wound dehiscence was significantly lower in the continuous skin suture group than in the interrupted skin suture group (RR 0.08; 95%CI 0.02-0.35).^7^

Considering the wide variety of suture materials regarding structural and chemical properties, the need for studies to guide the clinical choice of threads is undeniable, to provide the best possible tissue healing and recovery using quantitative and qualitative histological evidence. Therefore, in this study, we compare materials with different degradation-absorbable polyglycaprone 25 and nonabsorbable nylon-regarding their behavior in animal tissues sutured with both threads.

This study aims to comparatively evaluate Monocryl^®^ (polyglycaprone 25) and Ethilon^®^ (nylon) suture threads regarding the quality of healing favored by histological and immunohistochemical analysis of the scar tissue of rats subjected to sutures with both materials.

## METHODS

### Animals

In total, 12 adult Wistar rats, weighing about 320 g, from the vivarium of the Faculdade de Medicina do ABC (FMABC) were used. They were kept with food and water *ad libitum* in individual polypropylene boxes. The 12:12-hour light-dark cycle of the FMABC vivarium was also controlled. The humidity in the vivarium was about 70% and the room temperature about 23°C.

The rats were randomly divided into three groups of four animals each. Each group corresponded to a postsurgical evaluation date (one, seven, and 14 days):

Group 1 (one day after surgery):
  - Four rats subjected to four dorsal incisions and sutured with the following four types of threads;
Group 2 (seven days after surgery):
  - Four rats subjected to four dorsal incisions and sutured with the following four types of threads;
Group 3 (14 days after surgery):
  - Four rats subjected to four dorsal incisions and sutured with the following four types of threads.

The rats were anesthetized with ketamine (50 mg/kg i.p.) and xylazine (10 mg/kg i.m.), and then their backs were shaved. To demarcate the skin to be removed, a specially made metal punch with a cutting blade on its lower edge, similar to the tool used in plastic surgeries, was used. With this instrument, four 3-cm longitudinal incisions were made on the dorsal skin, with a distance of 2 cm between them, reaching the subcutaneous space. The incisions were made in the dorsal region of the rats so that they would not access the incision with their mouths. The rats received tramadol (10 mg/kg) intramuscularly immediately after the end of the surgery and every 12 hours for the first 24 hours after surgery to obtain analgesia. Due to the nature of the study, anti-inflammatory medications were not used. The rats received 0.2 mL of antibiotic (Veterinary Pentabiotic for Small Animals - Fort Dodge 2000 IU/mL) intramuscularly as a prophylactic measure in a single dose to prevent infection.

**Figure 1:**
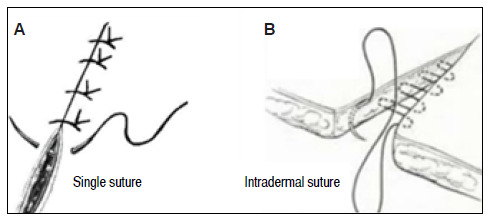
A: Representation of the single suture technique; B: Representation of the intradermal suture technique.

Hemostasis was performed by digital compression, using sterile gauze. Each rat was randomly subjected to one of the following types of suture (Figure 1):

- Single suture^3^ ^),(^ ^8^ ^),(^ ^9^ with Ethilon^®^ nylon;
- Single suture with Monocryl^®^;
- Interrupted intradermal suture^3^ ^),(^ ^8^ ^),(^ ^9^ with Monocryl^®^;
- Continuous intradermal suture with Ethilon^®^ nylon.

Immediately after surgery, the rats were placed in individual polypropylene boxes, receiving water and food *ad libitum* and analgesia with tramadol (10 mg/kg i.m.)-immediately after surgery and every 12 hours for the first 24 hours after surgery. All rats were examined daily for mobility and a macroscopic evaluation of the surgical wound was performed to assess the presence or absence of secretion, crusts, or necrosis. Data were evaluated and recorded on a specific, individual form for each rat.

The incisions were photographed by digital camera at pre-established periods (first, seventh, 14th, and 28th days) with protocol records for later comparison by digital planimetry (healing assessment by measuring the wound area) using the Image Tool software (University of Health Center, USA).

On the pre-established day for euthanasia (first, seventh, 14th, and 28th days), two rats from each group were weighed and received an overdose of sodium thiopental (100 mg/kg i.p.). Then, after confirmation of cardiopulmonary arrest and lack of reflexes, they were fixed on the operating table for tissue collection for morphological and molecular analysis. The wound was excised with a margin of 1 cm of intact skin around the incision, in depth to the muscle fascia. Each piece was individually identified, fixed in Styrofoam, and placed in a 10% formalin solution for slide preparation and histological evaluation.

Tissues were embedded in paraffin for histological sections 20 μm thick on a microtome.

The histological sections of the wound were stained using the hematoxylin-eosin and Picrosirius methods.

### Hematoxylin-eosin staining of histological sections

Initially, the histological sections of the wound were deparaffinized and hydrated. Then, hematoxylin staining was performed (15 to 20 minutes), followed by washing in running water for 10 minutes. The sections were then placed in 1% HCl alcoholic solution (1 mL of HCl in 99 mL of 70% alcohol) until the desired intensity was reached. The sections were quickly washed in running water, after differentiation, and then stained with eosin for two minutes and washed in running water until the water was clear. They were dehydrated, quickly in 70% alcohol, followed by 95% alcohol, 100% alcohol, and xylene, and, finally, the slides were covered with ERV-MOUNT to place a coverslip.

### Picrosirius staining of histological sections

After deparaffinization and hydration, the sections were stained in 0.1% Sirius red solution dissolved in saturated aqueous picric acid for one hour. Then, they were washed in running water (five minutes), counterstained with Ehrlich’s hematoxylin (two minutes), and washed again in running water (five minutes). After this process, the sections were dehydrated in an ethanol gradient, cleared in xylene, and mounted in Entellan^®^. The use of this stain, besides identifying collagen (which acquires an intense red color under conventional light), allows a qualitative assessment of the degree of collagen aggregation when analyzed under polarized light, according to Junqueira, Bignolas, and Brentani.^10^

### Verhoeff’s stain

After deparaffinization and hydration, the sections were stained with Verhoeff’s solution for 30 seconds. Then, they were carefully washed in distilled water. The sections were covered with wound chloride solution for 15 seconds and washed with distilled water, and the slides were covered with Van Gieson’s stain for three minutes. After this process, the sections were dehydrated in an ethanol gradient, cleared in xylene, and mounted in Entellan^®^. Verhoeff’s stain highlights the collagen fibers with a red or orange color and the presence of elastin with a black or blue color.

### Immunohistochemistry

The tissue fixed in 10% formalin, sectioned on the microtome, and 20-μm sections were separated for immunohistochemistry with specific antibodies to metalloproteinases 1, 2, and 9 and TIMP-1. The sections were initially washed with PBS (0.01 M; pH 7.4) for 15 minutes. Antigen exposure was performed in 10/1 mM Tris/EDTA buffer (pH 9.0) for five minutes, followed by heating in an oven at 70°C for 30 minutes in 10 mM sodium citrate buffer (pH 6.0). After washing with PBS for 15 minutes, endogenous tissue peroxidases were blocked with 1% hydrogen peroxide in PBS for 10 minutes. After washing again for 15 minutes, nonspecific binding sites were blocked for 60 minutes with a solution of normal goat serum and Triton X-100 (nonionic detergent) diluted in PBS. Then, without washing, but removing excess blocking solution, the sections were incubated for 24 hours at 4°C with their respective primary antibodies anti-metalloproteinases 1, 2, and 9 and TIMP-1 (Santa Cruz Biotechnology). Negative controls, omitting primary antibodies, were included in the processing of sections to avoid nonspecific labeling. The sections were then incubated with their respective peroxidase-coupled secondary antibodies and mounted on gelatinized slides, dried, and covered with coverslips using appropriate mounting medium for immunohistochemistry.

The research project was approved by the Animal Research Ethics Committee of the Faculdade de Medicina do ABC (CEUA-FMABC) on November 19, 2020, registration number 12/2020.

## RESULTS

The results from the first group of rats showed some open wounds, probably due to self-made scratches, and cellular evidence of an early healing/closing process of organized tissue.

**Figure 2:**
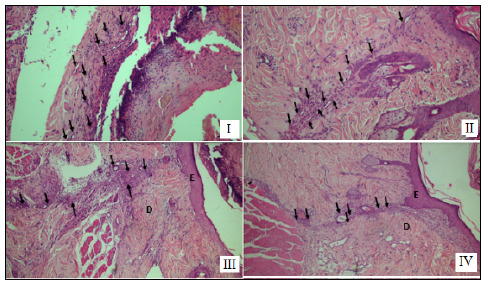
I: Simple suture and poliglycaprone 25 seven days after surgery; II: Simple suture with nylon seven days after surgery; III: intradermal suture with poliglycaprone 25 14 days after surgery; IV: intradermal suture with nylon 14 days after surgery.

Regarding the second group, all associations showed compatible granulation tissue. However, the single suture-nylon association caused a greater inflammatory reaction. In the intradermal suture-poliglycaprone association, we observed a more advanced fibrotic union. The intradermal suture-nylon association presented a better healing aspect (Figure 2).

D: dermis; E: epidermis.

At 14 days postoperatively, all associations in all rats showed compatible healing and no evident difference in the histological pattern of each healing.

## DISCUSSION

Several materials can be used for skin closure, with different techniques, in order to minimize complications such as scar pain, dehiscence, and infection, reduce surgical time, and improve aesthetics. Sutures with absorbable and nonabsorbable threads are the most widespread methods, as they are the simplest and least expensive techniques. In the literature, many studies compare different techniques and materials. However, the results are not unanimous.^11^

The results obtained in this study showed that:

- At the first day postoperatively (G1), the different suture techniques and materials showed no difference;
- At seven days postoperatively (G2), the rats subjected to nylon suture were at a more advanced stage of healing and the intradermal suture showed greater fibrotic union;
- At 14 days postoperatively (G3), we observed no evident difference in the histological pattern of each healing.

Normally, when a wound is closed with absorbable suture, the decrease in tensile strength in the first few weeks is gradual and linear. During this period, a leukocyte cellular response mounts to remove cellular debris and physical suture material, and this process overlaps with the second stage. Hydrolysis produces a lesser degree of tissue reaction compared with the enzymatic degradation process. In contrast, the in vivo tissue response around the nondegradable material involves fibroblasts encapsulating the suture to form a fibrous capsule. Adjacent macrophages and foreign body giant cells respond in a process known as frustrated phagocytosis, in which they attempt to enzymatically degrade the nondegradable suture.^12^

Our results are similar to the findings of Ribeiro et al.^13^ in 2005, in a clinical and histopathological analysis of the tissue reaction of nylon and polyglycaprone 25 monofilament threads in internal and external sutures in 40 rats. In their study, fibrosis formation was higher in external and internal sutures with polyglycaprone 25 from the seventh to the 21th day after surgery. The formation of granulation tissue, along with the presence of giant cells, was greater in external and internal sutures with nylon from the seventh day after surgery, since this material causes a greater inflammatory reaction, resulting in epithelialization of the suture path through the tissues with invagination of the wound edges.^13^

## CONCLUSION

We observed satisfactory wound healing with all associations. However, we highlight the considerably inflammatory reaction caused by the use of simple suture with nylon and the advanced wound healing seven days after surgery with the use of intradermal suture with poliglycaprone 25.
